# Right Bundle Branch Block Predicts Appropriate Implantable Cardioverter Defibrillator Therapies in Patients with Non-Ischemic Dilated Cardiomyopathy and a Prophylactic Implantable Cardioverter Defibrillator

**DOI:** 10.3390/diagnostics14111173

**Published:** 2024-06-01

**Authors:** Marta Jiménez-Blanco Bravo, Gonzalo Luis Alonso Salinas, Carolina Parra Esteban, Jorge Toquero Ramos, Miguel Amores Luque, Jose Luis Zamorano Gómez, Eusebio García-Izquierdo, Jesús Álvarez-García, Ignacio Fernández Lozano, Víctor Castro Urda

**Affiliations:** 1 Cardiology Department, Hospital Universitario Ramón y Cajal, Carretera de Colmenar Vieno km 9100, 28034 Madrid, Spain; martajbb@gmail.com (M.J.-B.B.); migueamo@hotmail.com (M.A.L.); zamorano@secardiologia.es (J.L.Z.G.); jalvarezg82@gmail.com (J.Á.-G.); 2 Centro de Investigación Cardiovascular en Red (CIBER-CV), Avenida Monforte de Lemos 3–5, 28029 Madrid, Spain; jorgetoquero@hotmail.com (J.T.R.); iflozano@secardiologia.es (I.F.L.); vcastrou14@yahoo.com (V.C.U.); 3 Cardiology Department, Hospital Universitario de Navarra (HUN-NOU), Calle de Irunlarrea 3, 31008 Pamplona, Spain; 4 Navarrabiomed (Miguel Servet Foundation), IdiSNA, 31008 Pamplona, Spain; 5 Heath Sciences Department, Universidad Pública de Navarra (UPNA-NUP), 31006 Pamplona, Spain; 6 Cardiology Department, Hospital Universitario Puerta de Hierro, Calle Joaquín Rodrigo 1, Majadahonda, 28222 Madrid, Spain; carolinaparraesteban@gmail.com (C.P.E.); usegij@gmail.com (E.G.-I.)

**Keywords:** non-ischemic dilated cardiomyopathy, implantable cardioverter defibrillator, sudden cardiac death, primary prevention, right bundle branch block

## Abstract

**Background:** The benefit of prophylactic implantable cardioverter defibrillators (ICDs) in patients with severe systolic dysfunction of non-ischemic origin is still unclear, and the identification of patients at risk for sudden cardiac death remains a major challenge. **Aims/Methods:** We retrospectively reviewed all consecutive patients with non-ischemic dilated cardiomyopathy (NICM) who underwent prophylactic ICD implantation between 2008 and 2020 in two tertiary centers. Our main goal was to identify the predictors of appropriate ICD therapies (anti-tachycardia pacing [ATP] and/or shocks) in this cohort of patients. **Results:** A total of 224 patients were included. After a median follow-up of 51 months, 61 patients (27.2%) required appropriate ICD therapies. Patients with appropriate ICD therapies were more frequently men (87% vs. 69%, *p* = 0.006), of younger age (59 years, (53–65) vs. 64 years, (57–70); *p* = 0.02), showed more right bundle branch blocks (RBBBs) (15% vs. 4%, *p* = 0.007) and less left bundle branch blocks (LBBBs) (26% vs. 47%, *p* = 0.005) in the ECG, and had higher left ventricular end-diastolic (100 mL/m^2^, (90–117) vs. 86, (71–110); *p* = 0.011) and systolic volumes (72 mL/m^2^, (59–87) vs. 61, (47–81), *p* = 0.05). In a multivariate competing-risks regression analysis, RBBB (HR 2.26, CI 95% 1.02–4.98, *p* = 0.043) was identified as an independent predictor of appropriate ICD therapies. **Conclusion:** RBBBs may help to identify patients with NICM at high risk of ventricular arrhythmias and requiring ICD intervention.

## 1. Introduction

In patients with left ventricular (LV) systolic dysfunction, approximately 50 to 65% of the total annual death rate is attributed to sudden cardiac death (SCD) [1], which is potentially preventable with an implantable cardioverter defibrillator (ICD). Several randomized trials have proven the efficacy of ICDs in primary prevention in this group of patients [2], and according to 2016 European Heart Failure (HF) guidelines, it was a class I indication to implant an ICD in patients with New York Heart Association (NYHA) functional class II or III symptoms and a left ventricular ejection fraction (LVEF) of 35% or less despite at least 3 months of optimal medical therapy (OMT), irrespective of etiology [3].

However, the benefit of ICD implantation in patients with non-ischemic HF has been questioned in the past years, particularly after the DANISH trial failed to demonstrate a reduction in all-cause mortality in these patients [4]. This led to a downgrade in the most recent European HF guidelines [5], and ICDs in primary prevention are now a class IIa indication in patients with non-ischemic etiology. Despite the outcome of the aforementioned trial, subgroup analyses revealed that younger patients and those with lower NT-proBNP did benefit from ICDs. There is clearly an unmet need for an improved risk stratification approach to guide the selection of patients for ICD implantation, particularly for those with non-ischemic dilated cardiomyopathy (NICM). 

The aim of our study was to identify predictors of appropriate ICD therapies, including anti-tachycardia pacing (ATP) and shocks, in a cohort of patients with NICM. Secondary endpoints included any inappropriate therapies or ICD-related infections.

## 2. Methods

### Patient Population and Study Design

We retrospectively reviewed all consecutive patients with NICM who underwent prophylactic ICD implantation between January 2008 and January 2020 in two tertiary centers. According to the ESC guidelines [3], all patients with symptomatic NICM, LVEF ≤ 35%, and at least 3 months of OMT were presented in a multidisciplinary session and subsequently accepted for ICD implantation if no major contraindications were found (e.g., life expectancy of less than one year due to non-cardiac reason). In addition, asymptomatic patients with LVEF ≤ 30% were also considered for this purpose. Ischemic cardiomyopathy, defined as the presence of significant lesions in any of the three main coronary arteries, was ruled out by coronary angiography or coronary computed tomography angiography. The diagnosis of idiopathic dilated cardiomyopathy was stablished after the detectable causes of HF were excluded, according to recent publications [6]. 

All ICD implants were supervised by three experienced electrophysiologists and were performed under local anesthesia, via right or left subclavian vein and with fluoroscopic guidance. The type of device was chosen according to the patient’s characteristics and the guidelines available at that time. All patients underwent a chest X-ray prior to discharge, to confirm adequate ICD lead placement, and an exhaustive check-up was performed to assure the correct functioning of the device. Ventricular tachycardia (VT) and ventricular fibrillation (VF) zones and therapies were programmed individually for each patient. All shocks were programmed to a maximum output of 30–40 J.

## 3. Study Variables, Follow-Up, and Outcomes

Demographics, clinical history, ECG, laboratory blood tests, imaging data including echocardiogram, cardiac magnetic resonance (MRI) and coronariography, baseline medications, and type of ICD were retrieved from electronical medical records by two independent investigators. The follow-up data were obtained from the outpatient visits every 6 to 12 months or from the event reports. The last registered visit at the outpatient clinic was considered as the last day of follow-up. Follow-up ended when the device was removed due to one of the following events: death, heart transplant, or explant with no subsequent replacement. The patients who underwent ICD replacement due to end of battery or device infection but had another ICD implanted immediately after were not considered lost to follow-up, and their data were analyzed considering the information registered in all their previous and current devices. The reported events were reviewed by an ad hoc committee.

The primary endpoint was the incidence of appropriate ICD therapies, defined as anti-tachycardia pacing (ATP) and/or appropriate shocks. Appropriate shocks were defined as those that terminated VT/VF and those not spontaneously terminated or could not be terminated by ATP. Ventricular arrhythmias were defined as any episode of ATP-terminated VT/VF. Registered non-sustained ventricular tachycardias or supraventricular tachycardias (whether treated or not) were not considered for this purpose. Secondary endpoints included any inappropriate therapies or ICD-related infections. Inappropriate shocks included those delivered in unnecessary situations (e.g., supraventricular tachycardia or due to lead problems). Device infection included ICD-related infectious endocarditis (IE) and local device infection, defined as an infection limited to the pocket of the cardiac device. Local device infection was clinically suspected when signs of inflammation at the generator pocket were present, including erythema, warmth, fluctuance, wound dehiscence, erosion, or purulent drainage [7,8].

All stored electrograms of episodes triggering ICD therapies were reviewed by two experienced electrophysiologists and were classified as appropriate or inappropriate according to the previously described criteria. 

The investigation conforms to the principles outlined in the Declaration of Helsinki. The study was approved by the local ethics committee, and informed consent was waived. 

### Statistical Analysis

Categorical variables are presented as percentages and compared using the chi-square test or Fisher’s exact test when appropriate. Continuous variables are presented as the mean ± standard deviation (SD) or median and interquartile range (IQR), depending on their distribution; *t* test and Wilcoxon rank-sum test were used to compare differences in these variables. All the tests were two-sided, and a *p* value of < 0.05 was used to determine statistical significance.

A multivariate competing-risks regression model was used to adjust for any confounding variables with a *p* value of < 0.05 in the univariate analysis, as well as LVEF, because it is the current parameter used to decide ICD implantation according to guidelines. Those variables with a percentage of missing data of more than 25% were not included. Kaplan–Meier curves with log rank statistics were calculated to analyze time to first ATP or shock. A sub-analysis of the cardiac resynchronization therapy (CRT) population was also prespecified. Statistical analysis was performed using the STATA software v15.1 (Stata Statistical Software, Release 15 2017; StataCorp LP, College Station, TX, USA).

## 4. Results

### 4.1. Clinical Characteristics of the Study Population

A total of 224 patients were included. Median age was 62.7 years (IQR: 55.1–69.0), and 73.7% were men. Most of patients were in the NYHA class II–III (88.7%). The most frequent cause of HF was idiopathic (*n* = 133, 59.8%), followed by alcohol-induced cardiomyopathy (*n* = 32, 14.3%) and familial cardiomyopathy (*n* = 14, 6.3%). Baseline treatment included betablockers (*n* = 207, 92.8%), angiotensin-converting enzyme inhibitors (ACEi) (*n* = 196, 87.5%) or Sacubitril–Valsartan (*n* = 17, 7.6%), and mineralocorticoid receptor antagonists (MRA) (*n* = 160, 71.4%). 

The median LVEF was 28% (IQR: 22–32), the median left ventricular end-diastolic volume (LVEDV) was 90.9 mL/m^2^ (IQR: 72.6–113.5), and 81 patients (36.2%) had moderate or severe mitral regurgitation. The mean left atrial (LA) diameter was 46 ± 9 mm. Baseline ECG revealed sinus rhythm in 143 patients (63.8%), atrial fibrillation or atrial flutter in 64 (28.6%), and ventricular pacing in 14 (6.3%). A total of 93 patients (41.5%) had a left bundle branch block (LBBB) pattern, 16 (7.1%) had a right bundle branch block (RBBB), and 23 (10.3%) showed a non-specific intraventricular conduction delay (IVCD). The median NT-proBNP prior to implantation was 1421.5 pg/mL (IQR: 503–4586). A minority of patients underwent CMR (*n* = 69, 30.7%), and of those, 42% (*n* = 29) showed some degree of cardiac fibrosis. The most implanted device was ICD-CRT (*n* = 116, 51.8%), followed by single-chamber ICD (*n* = 98, 43.8%) and dual-chamber ICD (*n* = 10, 4.5%). Baseline characteristics of patients are summarized in Table 1. 

### 4.2. Follow-Up and Outcomes 

After a median follow-up of 51 months (IQR: 26.8–77), 61 patients (27.2%) met the criteria for the primary endpoint: 35 (15.6%) received, at least, one appropriate shock, and 26 (11.6%) received ATP therapy. In comparison with patients without appropriate therapies, those that received ICD therapies were more frequently men (86.9% vs. 68.7%, *p* = 0.006), of younger age (median age: 58.7 years, IQR: 53.0–64.8 vs. 63.7, IQR: 57.0–69.8; *p* = 0.02), and had higher LVEDV, LVESV, and LA diameter. A non-statistically significant trend towards a lower LVEF in these patients was also noted. Regarding ECG, patients who required ICD intervention frequently had more RBBBs (14.8% vs. 4.3%, *p* = 0.007) and lesser LBBBs (26.2% vs. 47.2%, *p* = 0.005) than those in patients who did not undergo ICD therapies. 

Upon observing these results, a post hoc analysis was conducted to evaluate whether right ventricular impairment was the cause of this finding. Therefore, right ventricular function was assessed using tricuspid annular plane systolic excursion (TAPSE), obtained from the baseline echocardiogram of the patients. The result indicated that, among the 50 patients for whom this variable was measured or could be measured retrospectively, the results were similar in patients with and without appropriate therapies (19.6 vs. 19.5; *p* = 0.9549).

On the other hand, 18 patients (8.0%) presented device-related adverse events. Device infection occurred in 11 patients (4.9%), which resulted in ICD removal in 10 of them and 1 received intravenous antibiotic therapy and underwent a heart transplant before the device was removed. All patients received intravenous and oral antibiotic therapies according to the current recommendations (Table 2). In addition, seven patients (3.1%) received a total of 10 inappropriate shocks: 8 of the shocks were due to atrial fibrillation with an accelerated heart rate, 1 was due to sinus tachycardia, and 1 was due to lead fracture.

Finally, 43 patients (19.2%) died, 9 (4%) underwent heart transplant, and 5 (2.2%) underwent explant with no subsequent replacement. Of these five patients, one died at home due to SCD while waiting for the implant, two were not candidates for a new ICD because their LVEF had improved, and two had the device downgraded to CRT-P and single-chamber pacemaker due to advanced age and severe Alzheimer’s disease, respectively. 

### 4.3. Predictors of Appropriate ICD Therapies

**Figure 1:**
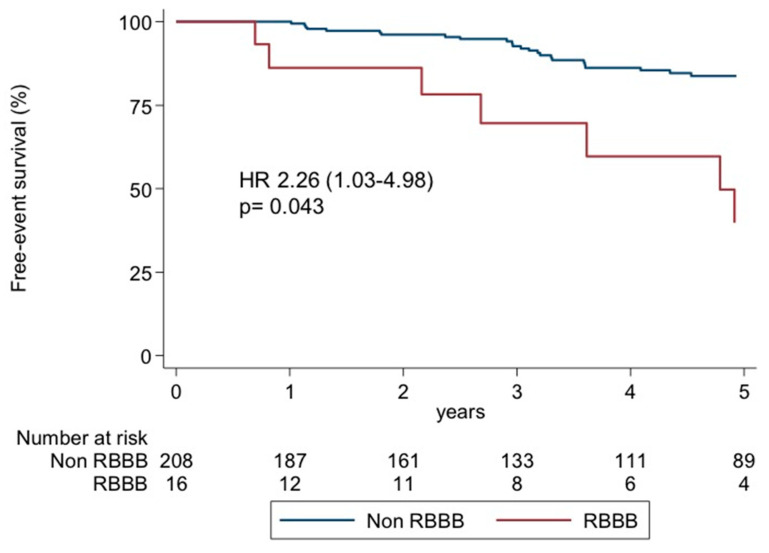
Kaplan–Meier survival curves for the primary endpoint according to the presence of RBBBs. RBBBs, right bundle branch blocks.

After a multivariate competing-risks regression analysis adjusted for age, sex, RBBB, LBBB, and LVEF, the only independent predictor of ATP or appropriate shocks was the presence of RBBBs at baseline (HR 2.26, CI 95% 1.03–4.98; *p*= 0.043). Table 3 shows the complete regression model. Figure 1 shows the Kaplan–Meier survival curves for the primary endpoint according to the presence of RBBBs.

A sub-analysis with multivariate competing risks of the CRT population (*n* = 116) was also performed, but the presence of RBBBs was not statistically significant in this subset of patients (HR: 2.18, CI 95%: 0.71–6.71, *p* = 0.173). 

## 5. Discussion

### 5.1. Main Findings

In a contemporary multi-center cohort of patients with NICM and a prophylactic ICD implantation, around one in four presented appropriate therapies after a median follow-up of more than 4 years, while a minority had complications related to the device. In addition, RBBB was independently associated with a higher risk of ventricular arrhythmias requiring ICD intervention. 

### 5.2. Primary Prevention of Sudden Cardiac Death in NICM

Many studies have been published on ICDs as a primary prevention in patients with NICM, with heterogenous results. The CAT and the AMIOVIRT studies both failed to show a difference in mortality between ICD and OMT or amiodarone after 5 or 3 years of follow-up, respectively [9,10]. In the DEFINITE trial, there was a trend towards lower all-cause mortality in the ICD group, and a statistically significant difference was observed for sudden deaths from arrhythmia [11]. In the SCD Heft, ICD implantation was associated with a 23% reduction in overall mortality. However, when subgroup analyses were performed, the difference between ICD therapy and placebo in non-ischemic HF was found to be non-significant [12]. More recently, in 2016, the DANISH trial concluded that ICD implantation in patients with symptomatic systolic heart failure of non-ischemic origin was not associated with a significantly lower long-term rate of death from any cause [4]. 

All these trials were summarized in a metanalysis, which included 2967 patients with NICM. The use of ICD was associated with a significant reduction in total mortality (HR: 0.78, *p* = 0.003), as well as arrhythmic death (HR: 0.46, *p* = 0.0005). However, the benefit in terms of total mortality was only seen in younger patients (<65 years) and in those without CRT [2]. Our event rate is similar to the ones reported in previous studies, which approximately range from 23 to 42% [13,14].

Taking all this into account, it is clear that there is at least a subgroup of patients with an HF of non-ischemic origin that would benefit from ICD implantation as a primary prevention. The method to identify this subset of patients remains unclear. 

### 5.3. Identifying High-Risk Patients for Sudden Cardiac Death 

To our knowledge, this is the first study that reports a relation between RBBBs and malignant ventricular arrhythmias. Lai et al. reported a similar incidence of RBBBs in a cohort of hospitalized patients with idiopathic dilated cardiomyopathy (7.3%), and they conclude that RBBB was an independent predictor of all-cause mortality in these patients [15]. However, they did not report its possible association with ICD nor described the incidence of lethal arrhythmias. Additionally, Li et al. reported that the presence of RBBBs or IVCDs was an independent predictor of all-cause mortality in patients with dilated cardiomyopathy, with a similar incidence of RBBBs (7.3%) [16].

One possible explanation for this association is that the presence of RBBBs might imply RV dysfunction and, thus, a more advanced stage of non-ischemic cardiomyopathy. Data supporting this hypothesis include a recent sub-analysis of the DANISH study, which revealed that ICD implantation significantly reduced all-cause mortality in patients with RV systolic dysfunction (determined by cardiac magnetic resonance), but not in patients with preserved RV function [17]. However, they do not specify whether these patients had a higher incidence of RBBBs. In our analysis, we were unable to find an association between RV function and the presence of RBBBs. However, since this was not a pre-specified objective of the study, there was significant information loss in this regard. Therefore, we can only hypothesize that measuring it as a potential confounding factor in future studies would be useful.

Male sex has already been identified as a possible factor associated with ICD therapies. In the DEFINITE trial, the risk of ICD shock was 2.56 times lower in women [11], and a prospective study including 657 patients with non-ischemic heart disease found that male patients were more likely to die or receive appropriate ICD therapies during a median follow-up of 3.3 years (HR: 0.76, *p* = 0.03) [18]. It is well known that men have a higher rate of ventricular arrhythmias and SCDs [19], although the reason for this gender difference remains unclear. Some studies have suggested differences in cardiac repolarization or hormonal influence, but the relation with arrhythmogenic risk has not been clearly established. 

Younger age has also been suggested as a possible criterion for patient selection in several studies. A sub-analysis of the DANISH trial found that the optimal age cutoff for ICD implantation in primary prevention was presently ≤70 years of age [20]. This may be explained by the fact that the rate of non-sudden cardiac death is much higher in the older population. In our study, patients with appropriate therapies were significantly younger, but this interaction was lost in the multivariate analysis. 

## 6. Complications Related to ICD Implantation

Side effects associated with device implantation are not negligible. Our series reports an incidence of device infection of 4.9%, similar to the one described in recent studies [4], and although our rate of inappropriate shocks was only 3.1%, it was still significant. Inappropriate shocks have been progressively reduced over the past few years due to the use of less aggressive ICD settings, but it is still one of the main setbacks to ICD implantation in primary prevention.

### Limitations

Even though it is a retrospective study, the fact that all patients that fulfilled the ESC guideline criteria and underwent ICD implantation can mitigate this aspect. Secondly, our median follow-up is limited to 51 months, and although most studies regarding ICD in primary prevention have a shorter follow-up, it is possible that some of the events of interest (appropriate or inappropriate shocks, ATP, or device infection) could have occurred later on. Lastly, only a minority of patients underwent CMR in our study (*n* = 69, 30.7%), and this limited our ability to relate the presence of late gadolinium enhancement to ventricular arrhythmias or appropriate shocks. Similarly, RV function was measured in only 50 patients (22.3%), which does not allow us to determine if this is a confounding factor for the obtained results.

## 7. Conclusions

In a contemporary multi-center cohort of patients with NICM and a prophylactic ICD implantation, around one in four presented appropriate therapies after a median follow-up of more than 4 years, while a minority had complications related to the device. The presence of right bundle branch block at baseline was the only independent predictor of ATP or appropriate shocks, and it may help to identify patients with non-ischemic dilated cardiomyopathy at high risk of ventricular arrhythmias and requiring ICD intervention.

## Figures and Tables

**Table 1:** Baseline characteristics of the cohort.

	Total (*n* = 224)	No Therapy (*n* = 163)	Appropriate Therapies (*n* = 61)	*p*
Age, years (median, IQR)	62.7 (55.1–69.0)	63.7 (57.0–69.8)	58.7 (53.0–64.8)	**0.0204**
Male sex, *n* (%)	165 (73.7%)	112 (68.7%)	53 (86.9%)	**0.006**
Hypertension, *n* (%)	121 (54.3%)	88 (54.3%)	33 (54.1%)	0.976
Diabetes, *n* (%)	64 (28.8%)	50 (30.9%)	14 (23.3%)	0.271
Atrial fibrillation, *n* (%)	88 (39.5%)	62 (38.3%)	26 (42.6%)	0.553
NYHA class, *n* (%)				0.7961
I	20 (9.1%)	17 (10.7%)	3 (4.9%)	
II	117 (53.2%)	81 (50.9%)	36 (59.0%)	
III	78 (35.5%)	57 (35.9%)	21 (34.4%)	
IV	5 (2.3%)	4 (2.5%)	1 (1.6%)	
NT-proBNP, pg/mL, median (IQR)	1421.5 (503–4586)	1396 (501–4755)	1465 (515–4586)	0.9526
Coronariography, *n* (%)	210 (94.2%)	154 (95.1%)	56 (91.8%)	0.355
ECG—rhythm				0.9131
Sinus rhythm, *n* (%)	143 (63.8%)	108 (66.7%)	35 (59,3%)	
Atrial fibrillation/flutter, *n* (%)	64 (28.6%)	41 (25.3%)	23 (39.0%)	
Ventricular pacing, *n* (%)	14 (6.3%)	13 (8.0%)	1 (1.7%)	
ECG—conduction disturbance				
LBBB, *n* (%)	93 (41.5%)	77 (47.2%)	16 (26.2%)	**0.005**
RBBB, *n* (%)	16 (7.1%)	7 (4.3%)	9 (14.8%)	**0.007**
IVCD, *n* (%)	23 (10.3%)	17 (10.4%)	6 (9.8%)	0.896
None, *n* (%)	62 (27.7%)	40 (24.5%)	22 (36.1%)	0.086
Echocardiogram at baseline LVEF (%) (median, IQR) LVEDV, ml/m^2^ (median, IQR) LVESV, ml/m^2^ (median, IQR) LA diameter, mm (mean ± SD) Significant mitral regurgitation (moderate or severe), *n* (%)	28 (22–31.9) 90.9 (72.6–113.5) 65.2 (49.5–84.7) 45.7 ± 8.7 81 (36.2%)	29 (24.2–32.0) 86.0 (71.3–110) 60.9 (47.4–80.5) 44.8 ± 8.5 61 (37.4%)	26 (20–30) 100 (90–116.8) 72.2 (58.9–87.4) 48.2 ± 8.8 20 (32.8%)	0.0770 **0.0106** **0.0467** **0.0480** 0.520
Type of cardiomyopathy Idiopathic, *n* (%) Alcoholic, *n* (%) Others Familial, *n* (%) Valvular, *n* (%) Hypertensive, *n* (%)	133 (60.5%) 32 (14.6%) 29 (13.0%) 14 (6.4%) 9 (4.1%) 3 (1.4%)	98 (61.3%) 21 (13.1%) 22 (13.6%) 10 (6.3%) 6 (3.8%) 3 (1.9%)	35 (58.3%) 11 (18.3%) 7 (11.6%) 4 (6.7%) 3 (5.0%) 0 (0%)	0.9199
Heart failure medications ACE inhibitors, *n* (%) Betablockers, *n* (%) Mineralocorticoid-receptor antagonist, *n* (%) ARNI, *n* (%) Amiodarone, *n* (%)	196 (88.3%) 207 (92.8%) 160 (71.4%) 17 (7.7%) 24 (10.8%)	143 (88.8%) 153 (94.4%) 119 (73.5%) 15 (9.3%) 18 (11.4%)	53 (86.9%) 54 (88.5%) 41 (67.2%) 2 (3.3%) 6 (9.2%)	0.689 0.127 0.356 0.131 0.636
Type of implanted device Single-chamber ICD, *n* (%) Dual-chamber ICD, *n* (%) ICD-CRT *n* (%)	98 (43.8%) 10 (4.5%) 116 (51.8%)	70 (42.9%) 7 (4.3%) 86 (52.8%)	28 (45.9%) 3 (4.9%) 30 (49.2%)	0.655 0.924 0.657
Outcomes Death, *n* (%) Heart transplant, *n* (%) Death or heart transplant, *n* (%)	43 (19.2%) 9 (4.0%) 52 (23.2%)	27 (16.6%) 3 (1.8%) 30 (18.4%)	16 (26.2%) 6 (9.8%) 22 (36.1%)	0.102 **0.007** **0.005**

LBBB, left bundle branch block; RBBB, right bundle branch block; IVCD, intraventricular conduction delay; LVEDV, left ventricular end-diastolic volume; LVESV, left ventricular end-systolic volume; LA, left atrium; LVEF, left ventricular ejection fraction; MRI, magnetic resonance imaging; ACE, angiotensin-converting enzyme; ARNI, angiotensin receptor–neprilysin inhibitor; ICD, implantable cardioverter defibrillator; and CRT, Cardiac Resynchronization Therapy.

**Table 2:** Device infections.

Patient	Symptoms	Fever	Blood Cultures	ICD Cultures	Vegetation in TOE	Explant
1	Phlebitis	Yes	*Staphylococcus lugdunensis*	-	Yes	No
2	Urinary sepsis	Yes	*Negative*	Negative	Yes	Yes
3	Erythema	No	*Staphylococcus hominis*	*S. epidermidis* Acinetobacter	No	Yes
4	Missing data	No	Negative	*S. epidermidis* *S. hominis-hominis*	No	Yes
5	Wound dehiscence	No	Negative	*S. epidermidis*	No	Yes
6	Erythema and warmth	No	Negative	*S. epidermidis*	No	Yes
7	Wound dehiscence and purulent drainage	No	Negative	*S. aureus* *S. epidermidis*	No	Yes
8	Erythema, warmth and fluctuance	Yes	Staph coagulase negative	Negative	Yes	Yes
9	Erythema and fluctuance	No	Negative	*S. aureus*	-	Yes
10	Wound dehiscence, edema, and warmth	No	Not performed	*S. aureus*	-	Yes
11	Wound dehiscence and erythema	No	Negative	*S. epidermidis*	-	Yes

ICD, implantable cardioverter defibrillator; TOE, transesophageal echocardiogram.

**Table 3:** Multivariate competing-risks regression model.

	Adjusted Hazards Ratio	95% Confidence Interval	*p*
Age	0.99	0.97–1.02	0.645
Male sex	2.04	0.84–5	0.115
RBBB	2.26	1.03–4.98	**0.043**
LBBB	0.62	0.33–1.16	0.135
LVEF	0.99	0.96–1.03	0.626

RBBB, right bundle branch block; LBBB, left bundle branch block; and LVEF, left ventricular ejection fraction.

## Data Availability

The data can be requested from the first author or the corresponding author and will be provided upon reasonable request and adequate justification.
